# Disruption of *PTPS* Gene Causing Pale Body Color and Lethal Phenotype in the Silkworm, *Bombyx mori*

**DOI:** 10.3390/ijms19041024

**Published:** 2018-03-29

**Authors:** Xiaoling Tong, Pingfeng Liang, Songyuan Wu, Yuanhao Li, Liang Qiao, Hai Hu, Zhonghuai Xiang, Cheng Lu, Fangyin Dai

**Affiliations:** 1 State Key Laboratory of Silkworm Genome Biology, Key Laboratory of Sericulture Biology and Genetic Breeding, Agricultural Ministry, College of Biotechnology, Southwest University, Chongqing 400715, China; xltong@swu.edu.cn (X.T.); lpf230@163.com (P.L.); fly_jungle@163.com (S.W.); lyh.1860@163.com (Y.L.); qiaoliangswu@163.com (L.Q.); huhaiswu@163.com (H.H.); xbxzh@swu.edu.cn (Z.X.); 2 College of Biotechnology, Southwest University, Chongqing 400715, China

**Keywords:** silkworm model, *al^c^*, PTPS, BH4, phenylketonuria

## Abstract

Phenylketonuria (PKU) is an inborn error of metabolism caused by mutations in the phenylalanine hydroxylase (*PAH*) gene or by defects in the tetrahydrobiopterin (BH4) synthesis pathway. Here, by positional cloning, we report that the 6-pyruvoyl-tetrahydropterin synthase (*PTPS*) gene, encoding a key enzyme of BH4 biosynthesis, is responsible for the *al^c^* (albino C) mutation that displays pale body color, head shaking, and eventually lethality after the first molting in silkworm. Compared to wild type, the *al^c^* mutant produced more substrates (phenylalanine (Phe) and tyrosine (Tyr)) and generated less DOPA and dopamine. Application of 2,4-diamino-6-hydroxypyrimidine (DAHP) to block BH4 synthesis in the wild type effectively produced the *al^c^*-like phenotype, while BH4 supplementation rescued the defective body color and lethal phenotype in both *al^c^* and DAHP-treated individuals. The detection of gene expressions and metabolic substances after drugs treatments in *al^c^* and normal individuals imply that silkworms and humans have a high similarity in the drugs metabolic features and the gene pathway related to BH4 and the dopamine biosynthesis. We propose that the *al^c^* mutant could be used as an animal model for drug evaluation for BH4-deficient PKU.

## 1. Introduction

Phenylketonuria (PKU; OMIM 261600) is an inherent disorder of metabolism predominantly caused by mutations in the phenylalanine hydroxylase (*PAH*) gene, which results in the accumulation of phenylalanine (Phe). PAH associates with a native co-factor tetrahydrobiopterin (BH4) that hydroxylates Phe to tyrosine (Tyr) (Supplementary Figure S1A). Defects in either PAH or BH4 synthesis may result in hyperphenilalaninemia, which can cause intellectual disability when untreated [1].

Phenylketonuria may result in progressive irreversible neurological impairment during infancy and childhood when not detected and treated in a timely manner. The most common symptom is severe mental retardation, which is often coupled with a musty odor in their breath, skin, and urine, eczema, reduced melanin pigmentation, growth retardation, depressive and anxiety disorders, and epilepsy. The severity of the clinical phenotype is directly correlated to blood phenylalanine levels [2].

Neonatal screening for PKU is crucial, and because a PKU newborn shows no obvious clinical symptom, a continuous Phe-restricted diet can prevent intellectual disability [3,4]. However, in some BH4-defcienct PKU patients, this strategy has a limited role in impaired neuropsychological functioning, therefore, individuals are prone to depressive and anxiety problems [5]. The reason is that BH4 is not only a co-factor of PAH in the liver, but is also involved in cerebral neurotransmission by its co-factor and/or chaperone activities for Tyr hydroxylase (TH), tryptophan hydroxylase (TPH), and nitric oxide synthase [6,7,8] (Supplementary Figure S1B,C). Hence, more research is required to find a more thorough understanding and more efficient therapeutic strategy for the treatment of various clinical phenotypes in PKU patients.

The development of experimental animals is an important component in the study of human diseases. The silkworm, *Bombyx mori*, is a fully domesticated insect derived from the wild silkworm, *B. mandarina*, and has been used for silk production for about 5000 years [9]. The silkworm is dependent on humans for its survival. From the beginning of the 19th century, the silkworm has also been utilized as an animal model for scientific discovery in microbiology, physiology, and genetics. Through nearly 100 years of classical genetics, more than 600 mutant strains of the silkworm have been obtained and preserved in China and Japan. It was recently considered as a model organism for Lepidoptera, which is the second largest insect order, following the completion of its genome sequence. Today, the silkworm plays a role in three major areas: basic research, sericulture, and biotechnology [10].

**Figure 1:**
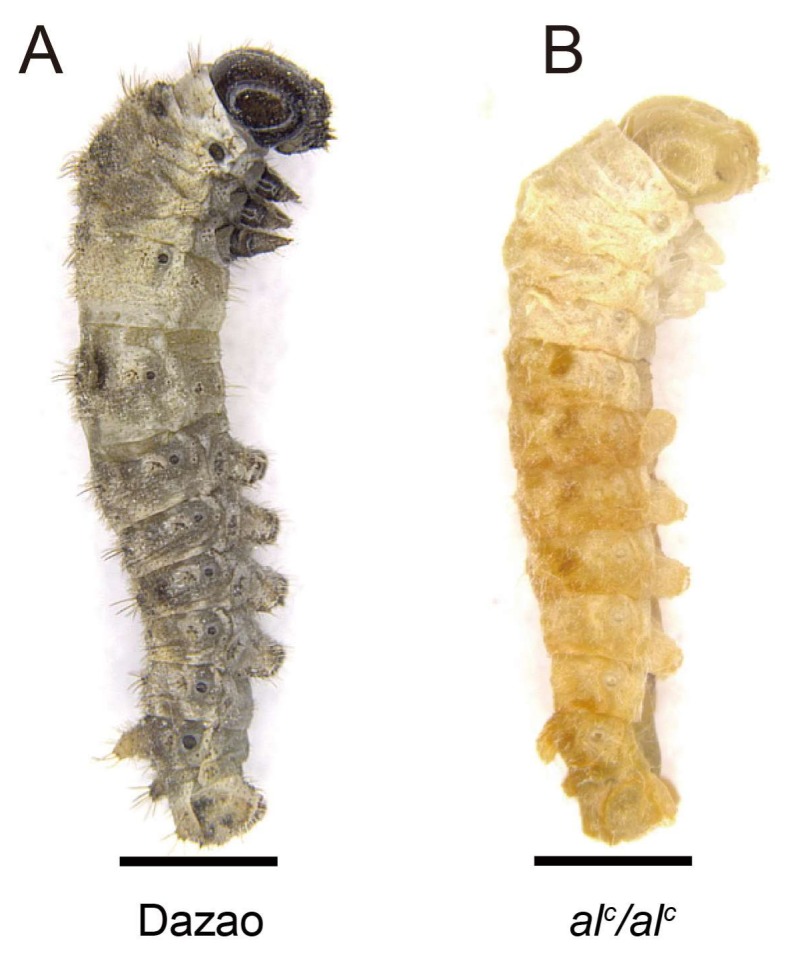
Phenotype of wild type (Dazao) and mutant albino C (*al^c^*). At the beginning of the second instar, the *al^c^* larvae showed a pale color in the epidermis and head (**B**) compared to the dark-brown color of the wild type Dazao (**A**). Scale bar: 1 mm.

Albino C (*al^c^*) is a spontaneous autosomal recessive larval stage mutant that is preserved in the silkworm gene bank of Southwest University, China. The body color of this mutant is normal dark-brown during the larval first instar, and turns pale after the first molting (Figure 1B). In contrast, the wild type (Dazao) shows normal pigmentation (Figure 1A). The mutants display head shaking behavior, and die within two days when untreated. Classic genetic research determined that this silkworm mutant was controlled by a single locus on chromosome 5. In the present study, in order to identify the candidate gene for *al^c^*, fine mapping was performed. In addition, we propose a molecular mechanism underlying this particular phenotype and potential application of this disease model.

## 2. Results

### 2.1. Fine Mapping and Gene Cloning

**Figure 2:**
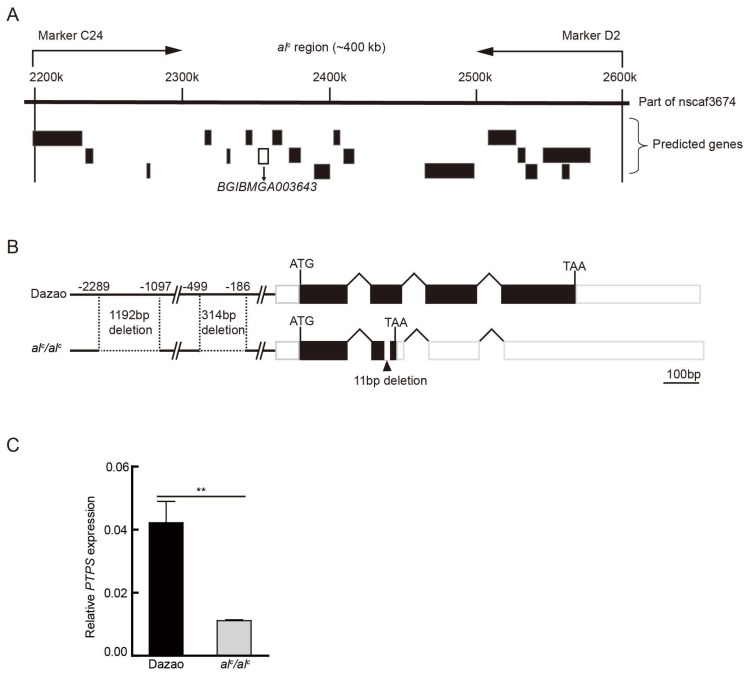
Positional cloning of the *al^c^* mutant. (**A**) Mapping of the *al^c^* locus on linkage group 5 using 170 BC_1_ (backcross, first generation) individuals. The *al^c^* locus was narrowed down between the PCR markers C24 and D2, by approximately 400 kb. Putative genes predicted by the Silkworm Genome Database are shown below the map, and *BGIBMGA003643* is shown in the black box. (**B**) The genomic structure of *BGIBMGA003643* in the wild type Dazao and *al^c^* mutant. A horizontal line, white box, black box, and polygonal lines indicate the upstream regulatory region, UTRs, ORFs, and intronic regions, respectively. In *al^c^*, an 11-bp deletion in exon 2 results in a premature stop codon. In addition, 1192-bp and 314-bp deletions were detected in the *al^c^* mutant in the upstream regulatory region. (**C**) Quantitative RT-PCR analysis of the relative expression levels of *PTPS* between Dazao and *al^c^* on the first day of the second instar (student’s *t*-test; *n* = 3, ** *p* < 0.01, data are expressed as the mean ± SD).

To identify the responsible gene of the *al^c^* mutant, genetic linkage analysis was performed. Owing to the lack of chromosomal recombination in female silkworms and the homozygous larval lethality of the albino C mutant, we selected F_1_ offspring from a cross between albino C (+/*al^c^*) female and Dazao (+/+) male. Ten Dazao and ten albino C mutants of BC_1_F (backcross female parent, first generation) were used in the cross, and the offspring were used for linkage analysis. In addition, 170 BC_1_M (backcross male parent, first generation) progeny (only *al^c^* individuals) from the male F_1_ backcrossed with albino C (+/*al^c^*), were used for recombination analysis. The *al^c^* locus was mapped within ~400 kb between markers C24 and D2 on Bm_nscaf 2674 (chromosome 5) (Figure 2A). This region encompasses 18 predicted genes in the Silkworm Genome Database (http://www.silkdb.org/silkdb/, SilkDB) [11]. The sequence of these predicted genes was used in a BLASTX search for their protein function in the non-redundant database (http://www.ncbi.nlm.nih.gov/) (Table S1). Because of the defective body color and larval lethalithy of *al^c^*, the candidate genes that were involved in pigment metabolism and lethality were primarily considered in the screening of the offspring.

**Figure 3:**
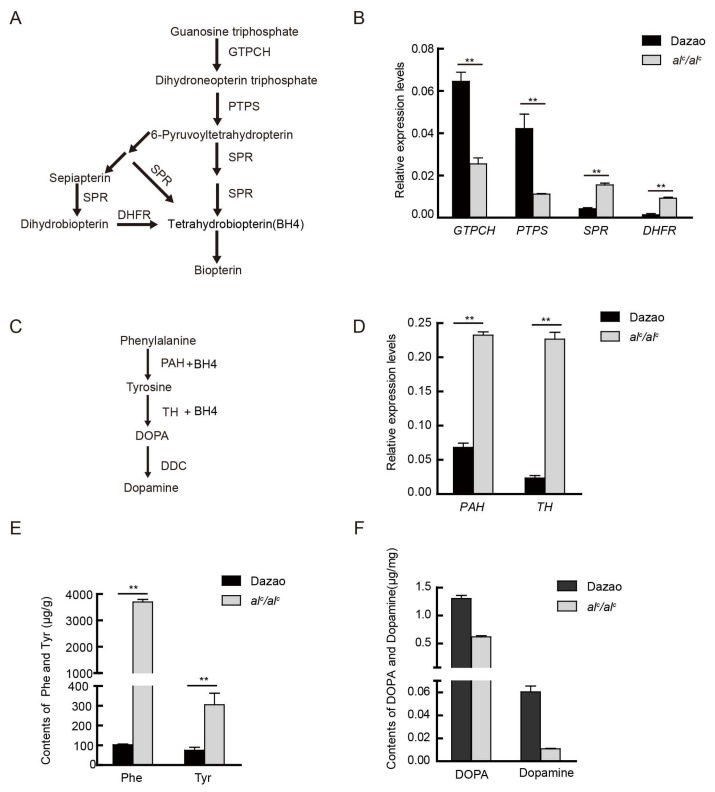
The comparison of related gene expression levels and material content in two pathways between Dazao and *al^c^* mutant. (**A**) The biosynthesis pathway of tetrahydrobiopterin (BH4) and pteridines from guanosine triphosphate (GTP). GTPCH: GTP cyclohydrolase; PTPS: 6-pyruvoyl-tetrahydropterin synthase; DHFR: dihydrofolate reductase; SPR: sepiapterin reductase. (**B**) Quantitative RT-PCR analysis of key genes during the biosynthesis pathway of BH4 at the beginning of the second instar between Dazao and *al^c^* (student’s *t*-test; *n* = 3, ** *p* < 0.01, data are expressed as the mean ± SD). (**C**) The biosynthesis pathway of melanin from phenylalanine. TH: tyrosine hydroxylase; PAH: Phenylalanine hydroxylase; DDC: DOPA decarboxylase. (**D**) Expression levels of the corresponding melanin genes between Dazao and *al^c^* at the beginning of the second instar (student’s *t*-test; *n* = 3, ** *p* < 0.01, data are expressed as the mean ± SD). (**E**) Amino acid analysis in the melanin pathway between Dazao and *al^c^* at the beginning of the second instar (student’s *t*-test; *n* = 3, ** *p* < 0.01, data are expressed as the mean ± SD). (**F**) Differences in DOPA and dopamine content at the beginning of the second instar between Dazao and *al^c^* (student’s *t*-test; *n* = 3, ** *p* < 0.01, data are expressed as the mean ± SD).

Based on the functional annotation of the 18 genes and the biological processes these may be involved in, the present study focused on one gene, *BGIBMGA003643*, which encodes 6-pyruvoyl-tetrahydropterin synthase (PTPS), a key enzyme in BH4 metabolism (Figure 2A). BH4 is an indispensable co-factor of PAH and TH. BH4 deficiency can interrupt dopamine biosynthesis, subsequently affecting melanin pigmentation [12]. We cloned the cDNA (complementary DNA) of the *PTPS* gene in the wild type Dazao and *al^c^*. In the wild type, the transcript of *PTPS* was 1014 bp in length, in which the open reading frame (ORF) was 504 bp in length and encoded a 167-amino acid protein. However, the transcript of *al^c^* has several single nucleotide polymorphisms (SNPs) and an 11-bp deletion in exon 2. This deletion, which results in a frame shift and a premature stop codon, was identified (Figure 3B), and the deletion transcript apparently produces a functionally null protein. Moreover, the genomic DNA of *PTPS* was cloned in *al^c^*, and a 314-bp and 1192-bp deletion were detected upstream of the transcriptional start site (Figure 2B). The qRT-PCR analysis determined that the *PTPS* was significantly downregulated in *al^c^* compared with that in Dazao at the beginning of the second instar (Figure 2C). *PTPS* was in complete linkage with the *al^c^* mutant, and the gene has important related physiological functions and significant sequence defects in mutants. These observations prompted us to consider *PTPS* as a candidate gene of *al^c^*.

### 2.2. Expression Levels of Key Genes Involved in the BH4 Pathway

BH4 is synthesized from guanosine triphosphate (GTP) through a cascade of three enzymes: GTP cyclohydrolase I (GTPCH), 6-pyruvoyl-tetrahydropterin synthase (PTPS), and sepiapterin reductase (SPR) (Figure 3A). PTPS is located upstream of the BH4 biosynthesis pathway. To clarify whether the inactivation of this enzyme affects the transcription levels of other key genes in this pathway, we investigated the expression levels of the *GTPCH* (AB439287), *SPR* (AK385894.1), and dihydrofolate ereductase (*DHFR*, JQ348842.1) genes in the Dazao and *al^c^* strains by qRT-PCR at the beginning of the second instar. The expression level of *GTPCH* was significantly downregulated, whereas that of *SPR* and *DHFR* were significantly upregulated in the *al^c^* strain compared to that in wild type Dazao (Figure 3B).

*PTPS* dysfunction causes the abnormal accumulation of its substrate, dihydroneopterin triphosphate, which apparently inhibits the expression level of *GTPCH*. Moreover, a lack of substrates (6-pyruvoyltetrahydropterin and dihydrobiopterin) induces the upregulation of *SPR* and *DHFR*. On the other hand, wild type Dazao exhibited moderate levels of *SPR* and *DHFR* expression. The observed abnormal expression of these genes in the BH4 biosynthesis pathway is suggestive of a disruption in BH4 biosynthesis.

### 2.3. The Expression Level of Key Genes and Content of Substances in the Melanin Metabolic Pathway

BH4 is not only a co-factor of PAH in the liver, but is also involved in cerebral neurotransmission by its co-factor and/or chaperone activities for Tyr hydroxylase. A comparison of the expression of key genes involved in the biosynthesis pathway of BH4 between Dazao and *al^c^* indicate that BH4 synthesis is blocked. Further, assessment of the expression level of *PAH* and TH, which are important genes of the dopamine metabolic pathway (Figure 3C; Supplementary Figure S2) showed that the two genes were significantly up-regulated in *al^c^* relative to that in Dazao (Figure 3D). Furthermore, content of Phe and Tyr, which are catalyzed by PAH and TH, respectively, were significantly higher in *al^c^* (36- and 4-fold higher, respectively) than that in Dazao (Figure 3E). In addition, the levels of DOPA and dopamine, which are the catalytic products of TH and important neurotransmitters and melanin precursors, were significantly lower (about 2.1- and 4.7-fold) in *al^c^* compared to that in the Dazao strain, respectively (Figure 3F). The *al^c^* mutant produced more substrates (Phe and Tyr) and enzymes (PAH and TH), but generated less DOPA and dopamine products; however, its deficiency in BH4 prevents them from synthesizing DOPA and dopamine. In addition, due to deficiencies in melanin precursors (DOPA and dopamine), the *al^c^* mutants are incapable of exhibiting normal melanin pigmentation.

### 2.4. Treatment Using a BH4 Inhibitor

In the *al^c^* mutant, a disorder involving BH4 biosynthesis can lead to an excessive accumulation of Phe, thereby reducing DOPA and dopamine levels, ultimately resulting in albinism. The present study applied a BH4 biosynthesis inhibitor (2,4-diamino-6-hydroxypyrimidine, DAHP) to wild type strain to create this PAH deficiency phenotype, and to investigate the biochemical metabolic mechanism underlying this condition.

**Figure 4:**
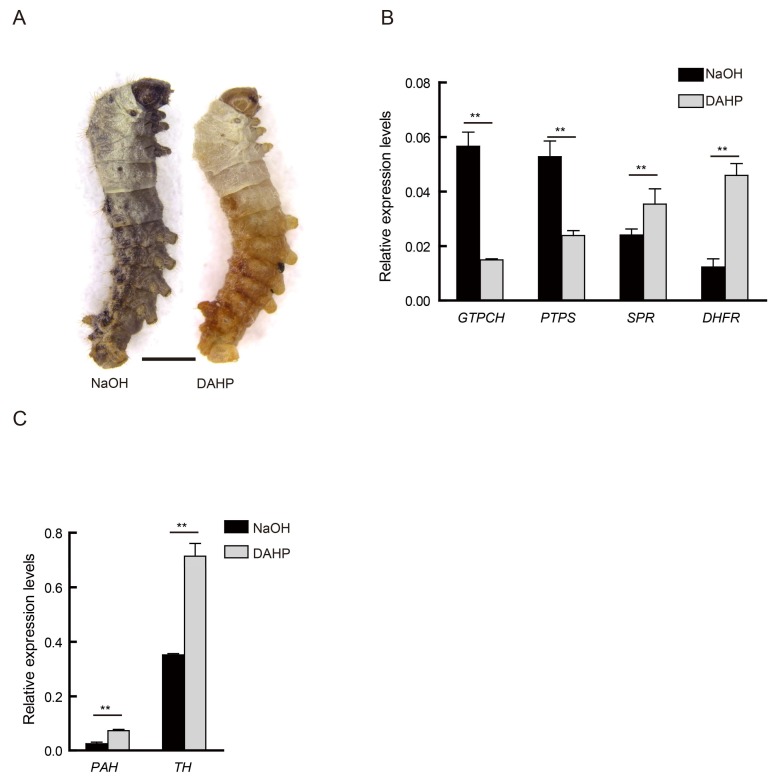
Oral administration of GTPCH inhibitor (2,4-diamino-6-hydroxypyrimidine (DAHP)). (**A**) The individual phenotype of treatment with GTPCH inhibitor, DAHP, and control group treatment with NaOH in the N4 strain at the beginning of the second instar. Scale bar: 1 mm. (**B**) The relative expression level of corresponding genes during the biosynthesis pathway of BH4 between DAHP-treated and NaOH-treated (control) at the beginning of the second instar (student’s *t*-test; *n* = 3, ** *p* < 0.01, data are expressed as the mean ± SD). (**C**) Differences in expression level of *PAH* and *TH* between DAHP-treated and NaOH-treated (control) at the beginning of the second instar (student’s *t*-test; *n* = 3, ** *p* < 0.01, data are expressed as the mean ± SD).

DAHP, a competitive inhibitor of GTPCH [13,14], was administered to newborn larvae of the wild type strain N4, and their phenotypic features were assessed at the beginning of the second instar (first molting). Strain N4 treated with low basic sodium hydroxide solution was used as control. Individuals showing extremely similar features as that of the *al^c^* mutants were observed in the treatment group at day 1 of the second instar (Figure 4A). Simultaneously, we detected the expression level of key genes in the BH4 and melanin pathways of these individuals. The results show that the expression levels of these genes in the *al^c^*-like individuals were similar to that in the *al^c^* mutants. The expression level of *GTPCH* and *PTPS* was significantly downregulated in the DAHP-treated group, whereas that of *SPR*, *DHFR*, *PAH*, and *TH* were upregulated (Figure 4B,C). These findings indicate that BH4 deficiency can lead to an albino phenotype, and the silkworm effectively responded to the drug treatment.

### 2.5. Treatment of BH4 Deficiency

Disorders in BH4 biosynthesis can lead to hypopigmentation, head shaking, and death in silkworm. We next determined whether the lethality and pale body color of the natural mutation (*al^c^*) and *al^c^*-like individuals induced by DAHP treatment could be rescued by BH4 supplement.

**Figure 5:**
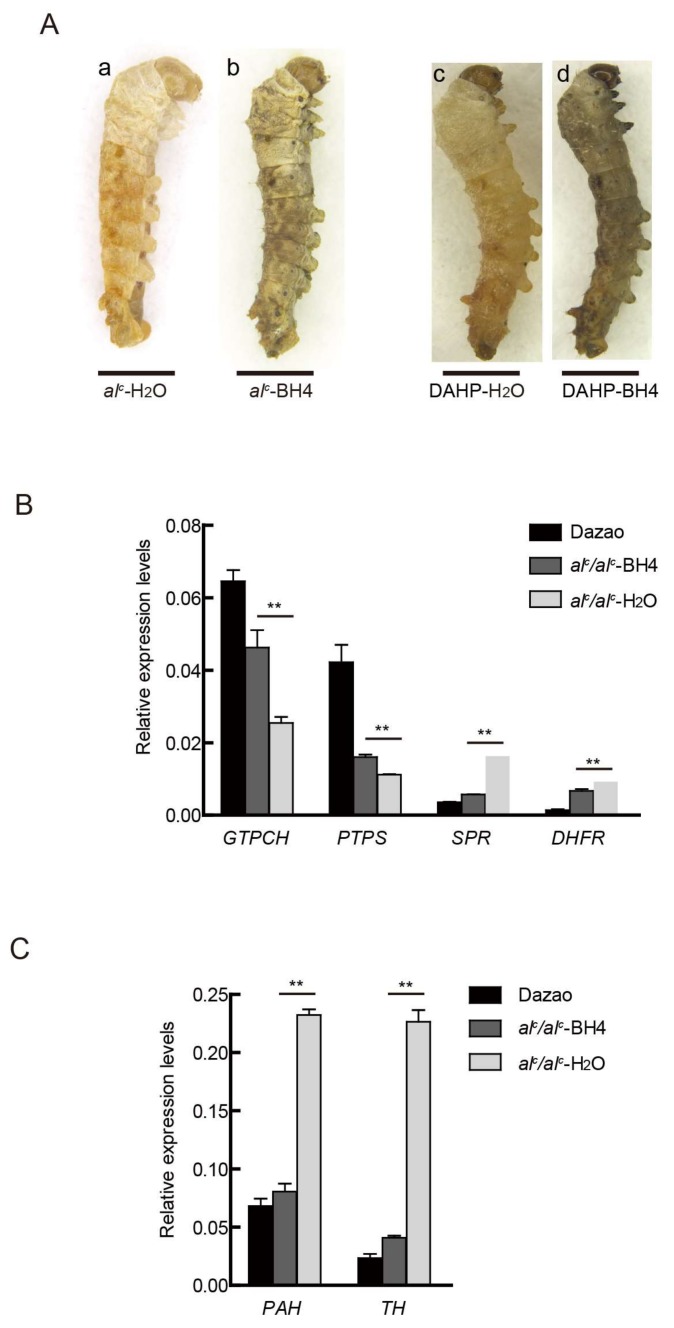
Rescue of the *al^c^* larvae by BH4 administration. (**A**) Phenotype of *al^c^* and DAHP-treated larvae in 30 mM BH4 treatment on day 2 of the second instar. Scale bar: 1 mm. (**B**) Expression differences in BH4 synthesis-related genes between Dazao and *al^c^* at day 2 of the second instar (student’s *t*-test; *n* = 3, ** *p* < 0.01, data are expressed as the mean ± SD). (**C**) Expression levels of the melanin metabolism genes between wild type Dazao and *al^c^* mutant at day 2 of the second instar (student’s *t*-test; *n* = 3, ** *p* < 0.01, data are expressed as the mean ± SD).

Newly hatched larvae of the *al^c^* mutants and members of the DAHP treatment group received 30 mM of BH4, and their phenotypes were monitored for the next two days. The same volume of H_2_O was given to the control group. The BH4 treatment group showed melanization compared to that in the control group (Figure 5A), and an increase in survival rate. Meanwhile, feeding dopamine to newly hatched larvae also converted the *al^c^* phenotype to exhibit normal pigmentation and prolongation of their lifespan (data not shown).

In addition, we investigated the expression levels of key genes involved in melanin; BH4 pathways returned to their normal levels. In particular, the expression levels of *PAH* and *TH* significantly declined (Figure 5B). These results indicate that drug treatment can rescue the defective body color and significantly improve the survival ratio of diseased silkworm (*al^c^* and DAHP-treated individuals).

## 3. Discussion

In the present study, the responsible gene for larval pigmentation, *al^c^*, was determined to encode PTPS, which is a key enzyme of BH4 biosynthesis. The *al^c^* mutant and wild type strain showed no observable differences during the embryonic and larval early instars. However, the body color of the *al^c^* mutants switched from normal dark brown to pale at the beginning of the second instar. Furthermore, the *al^c^* mutants showed excessive accumulation of Phe and deficiencies in DOPA and dopamine for cuticular pigmentation, thereby leading to an insufficient supply of *N*-acetyl dopamine (NADA) and *N*-β-alanyldopamine (NBAD) for mouthparts sclerotization and larvae death after the first molting.

In humans, BH4 deficiency results in phenylalanine accumulation, which causes PKU if untreated. Over 50% of human BH4-deficient patients suffer from PTPS deficiency [15]. The silkworm PTPS protein shares 64% amino acid identities with human PTPS. Furthermore, oral supplementation of BH4 and neurotransmitter precursors to silkworm *al^c^* mutants rescued the defective body color, lethal phenotype, and phenylalanine metabolism, while, application of the competitive inhibitor DAHP to the wild type also effectively produced the BH4-deficient phenotype. Although the BH4 levels could not be measured directly in the above treatments due to the lack of standards, the detection of gene expressions and metabolic substances imply that silkworm *al^c^* mutant and human BH4-deficient PKU have a high similarity in the drug metabolic features and the BH4 biosynthesis pathway, as well as in the downstream dopamine metabolic pathway. Based on this, the *al^c^* mutant could be a potential disease model for human PKU.

Animal models have greatly enriched our understanding of the molecular mechanism and the therapies for disease. The silkworm is a domesticated economic insect and is one of the model organisms for basic research. It has a clear genetic background, short life span, numerous progeny, controllable living environment, easy experimental operation and feeding, mature gene editing technology, low-cost, no ethical restrictions, and a considerable number of genes that are homologous to human disease-associated genes [16]. Because of these advantages, silkworm has received increasing attention as a human disease model. Thus, many silkworm disease models have been established recently, such as Parkinson’s disease [17], human sepiapterin reductase deficiency [12], insulin-related human disease [16], and Hermansky–Pudlak syndrome [18]. In addition, previous reports regarding the application of silkworm in the screening of antimicrobial drugs [19], anti-diabetic drugs, and traditional Chinese medicine [20] have implied that very similar drug metabolic features and pharmacodynamics were shared between silkworm and mammals. In the present study, the epidermis color of the *al^c^* mutants changed obviously upon drugs treatment. Thus, the readily visible color variation is helpful to evaluate the effectiveness of novel PKU therapeutic drug candidates. In conclusion, we presented experimental evidence that silkworm the *al^c^* mutant has great potential as a suitable disease model for human BH4-deficient PKU.

## 4. Materials and Methods

### 4.1. Silkworm Strains

The *al^c^* mutant and wild type strains, Dazao and N4, were supplied by the silkworm gene bank in Southwest University, China. These were reared on fresh mulberry at 25 °C under 12 h light: 12 h dark. Second instar larvae were used unless otherwise mentioned.

### 4.2. DNA Extraction and Sample Preparation

Total genomic DNA of the parental strains and F_1_ individuals was extracted from the whole moth. Genomic DNA of individuals used for mapping was extracted from whole larvae at the beginning of second instar using DNAzol^®^ (Invitrogen, Carlsbad, CA, USA). Total RNA was isolated from Dazao and *al^c^* at day 0 of the second instar using TRIzol^®^ (Invitrogen), according to the manufacturer’s protocol. cDNA was synthesized from total RNA samples using random primers (N9), oligo (dT) primers, and a moloney murine leukaemia virus reverse transcriptase (Promega, Madison, WI, USA), according to the manufacturer’s instructions.

### 4.3. Fine Mapping of Albino C Locus

Two silkworm strains, Dazao (+/+) and albino C (*al^c^*/*al^c^*) were used for mapping. Owing to the lack of recombination in female silkworms and the lethality of the albino C mutant, we selected F_1_ offspring from a cross between albino C (+/*al^c^*) ♀ and Dazao (+/+) ♂. Ten Dazao and ten albino C mutants of BC_1_F progeny from the cross F_1_ ♀ × *al^c^* (+/*al^c^*) ♂ were used for linkage analysis, and 170 BC_1_M progeny (only *al^c^* individuals) from the cross of albino C (+/*al^c^*) ♀ × F_1_ (+/*al^c^*) ♂ were used for recombination analysis. For fine mapping, 17 polymorphic PCR markers on chromosome 5 identified between Dazao (+/+) and albino C (*al^c^*/*al^c^*) were first confirmed to be linked with the *al^c^* locus by using the BC_1_F progeny, and subsequently were used for recombination analysis in BC_1_M individuals. The molecular markers used in the linkage and mapping analysis are listed in Supplementary Table S2. This analysis required the silkworm 9× assembly genome database (http://www.silkdb.org/silkdb/, SilkDB) and BLAST (http://www.ncbi.nlm.nih.gov/).

### 4.4. Cloning of the PTPS Gene

Total RNA was extracted from Dazao and *al^c^* at the beginning of the second instar as earlier described. The primers for the full-length cDNA and genome of the candidate genes were designed according to the sequence in GenBank and silkworm genome database (SilkDB). PCR products were cloned into the PMD19-T vector (TaKaRa, Otsu, Japan) and then sequenced. The primers used for cloning are listed in Supplementary Table S2.

### 4.5. Quantitative RT-PCR Analysis

To identify differences in the BH4 synthesis-related gene and melanin metabolism-related genes between Dazao and *al^c^* mutant strains, total RNA was isolated using TRIzol^®^ (Invitrogen) from the second instar larva, and then reverse transcribed to cDNA. Quantitative RT-PCR was performed using the ABI Prism 7000 sequence detection system (Applied Biosystems, Forster City, CA, USA) with a SYBR Premix EX-Taq-kit (TaKaRa, Otsu, Japan) according to the manufacturer’s protocol. The primers for the *GTPCH* (AB439287), *PTPS* (KR703273), *SPR* (AK385894.1), *DHFR* (JQ348842.1), *PAH* (GU953670), and *TH* (AB439286) genes are listed in Supplementary Table S2. sw22934, a eukaryotic translation initiation factor 4A (DQ443290.1) of silkworm, was used as an internal control.

### 4.6. Chemicals

DOPA (D9628-5G), dopamine (H8502-5G), DAHP (D19206-25G), and BH4 (T4425-5 mG) were purchased from Sigma-Aldrich (St. Louis, MO, USA). The Phe and Tyr standards used for quantitative analysis were provided by the Key Laboratory in Sichuan Province, Institute of Animal Nutrition, and Sichuan Agricultural University, YaAn 625014, China.

### 4.7. Quantification of Amino Acids and Catecholamine

We selected the second instar at day 0 between wild type Dazao and *al^c^* mutant strains for amino acid and catecholamine content analysis. The free amino acids were extracted as follows: the samples were homogenized with 800 µL 0.1 M hydrochloric acid (HCl) in centrifuge tubes, then the homogenate was mixed for 15 min using ultrasonication followed by centrifugation (12,000× *g*, 4 °C). The supernatant (600 µL) was transferred to a new tube containing 600 µL of 10% sulfosalicylic acid, followed by further centrifugation (12,000× *g*, 4 °C for 15 min). The supernatants were transferred and filtered with 0.22-µm membranes. A Hitachi (Tokyo, Japan) L-8800 amino acid analyzer physiological fluid system (lithium system) was used for amino acid content analysis. DOPA and dopamine were extracted and quantified according to Koch’s method [21]. Agilent1260 Infinity HPLC (Santa Clara, CA, USA) and Symmetry Shield RP18 (5 μM, 4.6 × 250 mm, Waters Corp., Milford, MA, USA) columns were used for HPLC analysis. Compared to known standards, amino acids and catecholamine standards were identified based on retention times, as follows: Phe 56.33 min; Tyr, 53.35 min; DOPA 5.858 min; and Dopamine 8.226 min (Supplementary Figure S2A,B).

### 4.8. DAHP Feeding Experiments

DAHP (Sigma-Aldrich), an inhibitor of GTPCH, was fed to newly hatched wild type strain N4 larvae. Treatment group: 2.5 g of DAHP was dissolved in 0.1 M sodium hydroxide (NaOH), and the volume was adjusted to 150 mL. Artificial feed (50 g) was then added to the 150-mL DAHP solution, which was mixed uniformly. The mixed artificial feed was sterilized at high pressure (98 °C, 25 min). Control group: 50 g of artificial feed was added to 150 mL of 0.1 M NaOH. The solution was then mixed until fully uniform and sterilized at high pressure (98 °C 25 min). The treatment was administered orally to larvae once a day for three days.

### 4.9. BH4 Feeding Experiments

BH4 (30 mM) was evenly spread on the surface of the artificial diet and fed to the newly hatched larvae once every day. The F_2_ offspring are an inbred line of F_1_ (+/*al^c^*) (Theoretically, only 1/4 larvae were *al^c^* individuals from a batch). At the beginning of the second instar larvae, the *al^c^* individuals were selected for BH4 feeding, and then the phenotypes were observed at the second instar on day 2. BH4 was administered orally once a day for three days. H_2_O was evenly spread on the surface of the artificial diet and was fed once a day for three days to newly hatched larvae as a control.

## Supplementary Materials

Supplementary materials can be found at http://www.mdpi.com/1422-0067/19/4/1024/s1.

## Abbreviations

PKU	phenylketonuria
PAH	phenylalanine hydroxylase
BH4	tetrahydrobiopterin
PTPS	6-pyruvoyl-tetrahydropterin synthase
SilkDB	Silkworm Genome Database
GTPCH	guanosine triphosphate cyclohydrolase I
SPR	sepiapterin reductase
DHFR	dihydrofolate reductase
NADA	*N*-acetyl dopamine
NBAD	*N*-β-alanyldopamine
DAHP	hydroxypyrimidine
