# Supplementary material for: Disruption of PTPS Gene Causing Pale Body Color and Lethal Phenotype in the Silkworm, Bombyx mori
Source: Int J Mol Sci. 2018 Mar 29;19(4):1024. doi: 10.3390/ijms19041024 (PMC5979516; doi:10.3390/ijms19041024)
Supplement: Supplementary file 1 [file ijms-19-01024-s001.pdf]

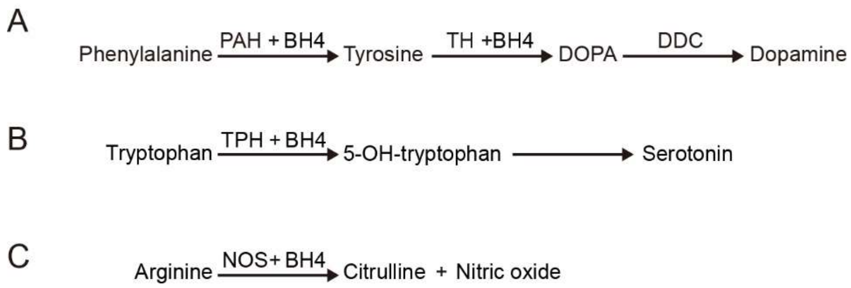

Supplementary Figure S1. BH4 is an important cofactor involved in dopamine, serotonin, and nitric-oxide synthase. PAH, phenylalanine hydroxylase; TH, tyrosine hydroxylase; TPH, tryptophan hydroxylase; NOS, nitric-oxide synthase.

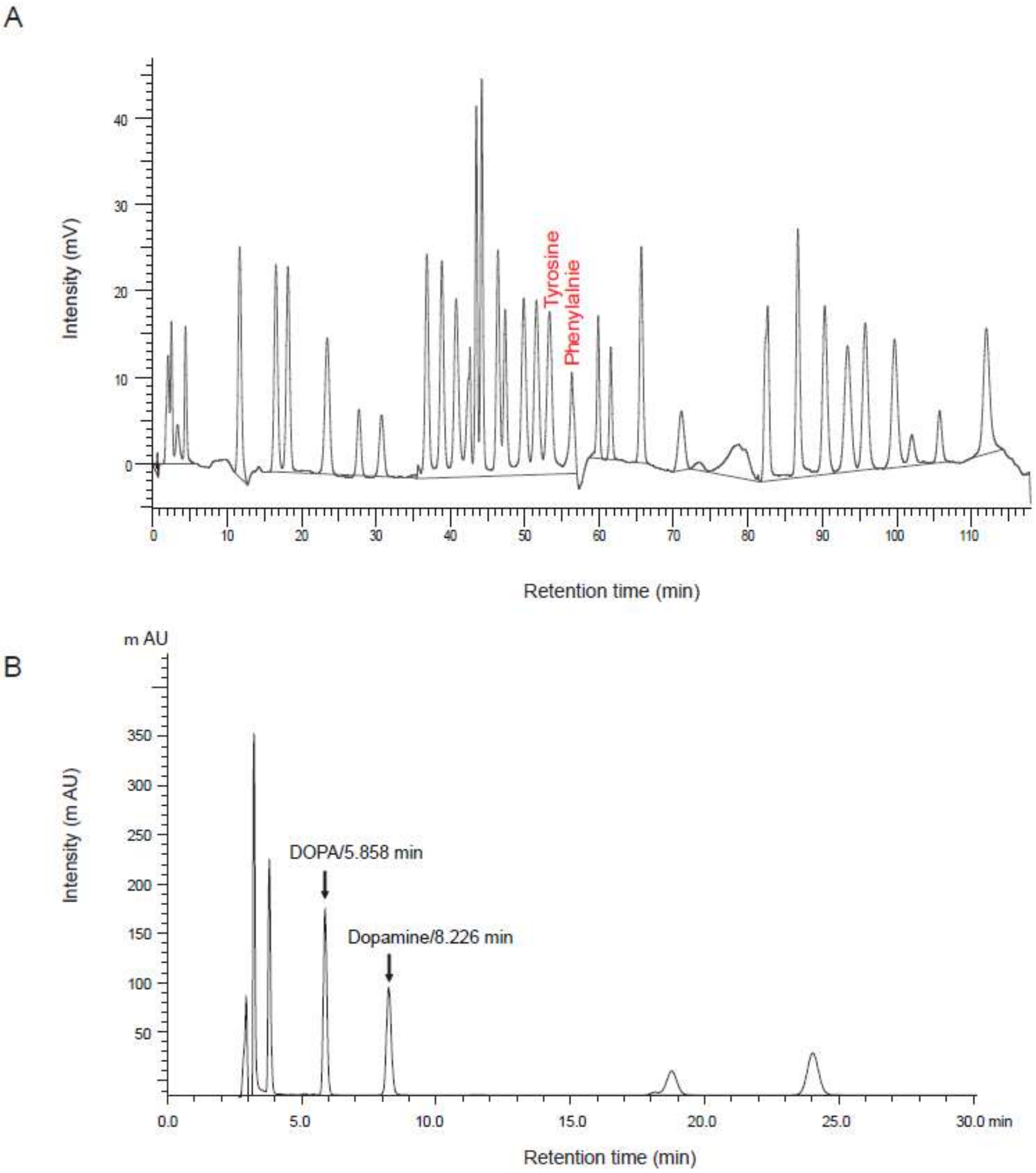

Supplementary Figure S2. Appearance time of standard sample of amino acid and catecholamines analysis. (**A**) Compared to known standards, amino acid standards were identified based on appearance times as follows: Phe 56.33 min; Tyr, 53.35 min, marked with red. (**B**) DOPA and dopamine appearance times are 5.858 min and 8.856 min, respectively.

Supplementary Table S1. Functional annotation of the genes in the mapping region.

Gene ID	Best BLAST hit in nr database; putative function	E-value	Identity
BGIBMGA003640	voltage-dependent calcium channel subunit α-2/Δ-3-like	0.0	92%
BGIBMGA003549	mitochondrial translational release factor 1	0.0	99%
BGIBMGA003548	unknown		
BGIBMGA003641	fork head domain-containing protein FD4-like	4e-170	100%
BGIBMGA003642	unknown		
BGIBMGA003547	fork head domain-containing protein FD4-like	4e-135	100%
BGIBMGA003643	6-pyruvoyltetrahydropterin synthase	1e-106	100%
BGIBMGA003546	alkaline ceramidase-like isoform X1	1e-153	83%
BGIBMGA003545	proteasomal ATPase-associated factor 1-like	0.0	79%
BGIBMGA003644	sorting nexin-17-like	0.0	95%
BGIBMGA003544	ubiquinol-cytochrome C reductase complex 14kD subunit	2e-50	99%
BGIBMGA003645	lipoyltransferase 1, mitochondrial-like isoform X1	0.0	100%
BGIBMGA003646	hypothetical protein	0.0	53%
BGIBMGA003647	tyrosine-protein kinase shark-like	0.0	100%
BGIBMGA003543	leucine-rich repeat-containing protein 70-like	0.0	100%
BGIBMGA003542	uncharacterized protein	4e-53	72%
BGIBMGA003648	ubiquitin-protein ligase E3C-like	0.0	98%
BGIBMGA003541	hypothetical protein	4e-169	46%

Supplementary Table S2. Primers used in this article.

Object	Primer Name	Sense Primer	Anti-sense Primer
For Mapping	S0512	ATTCTTTTCCACCTGATTGTTGTCT	CCATGTTCCGGGTGTCAGAG
	D2	GGTAACCGTATTAGCGACAAC	TAAGTAAATGCGATTAGTAGCG
	D3	CACGACCACCACGATTCTAT	CCAATTCCTATGATCCCAGTT
	D5	CGCCTTAGGTTACAGCAAGT	AATCGTCAGCTCCAAGTCAT
	D8	AGCCTTAGCACCAATGACTC	ATTAGTATTGAAGACGCTCTGG
	D11	CGGAAATAGGATGGACGACTT	CCATAGCGTGGTGATTGATTT
	D14	CTATCGGGATGGAATCTGCG	AATAGTCTCCAGTGGCGGTTG
	D16	CGTTTGCGTGTTGTCTCC	TTTGTTCTTCGCCCATCA
	D17	TAAAACCAGCCTTAGCACCAATGAC	TAAATCGTAAGACTTCCGTTGG
	D20	CACTGAACAGACAACCCAACT	CGTTTCCGATAACTGACCC
	D26	GTCGCATTCAGGTGTAGGT	ATGGATGGCGGAATAAAC
	C2	TCCCACTCCAACCGATAACTAC	CGATGCGATGAGCACTGAAT
	C10	CGTGTCGTTTGCGTAAGAGG	TTATGGTCGAGTGGTTCCAG
	C12	TTTACTGTTACGCAGAAGAATG	GTCTGGAGTAGTAGTCGTGCTG
	C13	GTTCACCTTCTGCTGCCCTTAT	CCCATCGTATCGCACTTTGT
	C16	GTGTTGCGACTGAGCGAGAT	CCTCCACGAATACGCTAAATG
	C24	TTCACATACGAGATACCAGGAG	GGCTCTAAAGTTGCGGTAAT
For Cloning	P1	ATTGGTCTTGGCGTAGTCCTC	TTTCCTCACCGATATTCTTGTT
	P2	CGTGTCGTTTGCGTAAGAGG	TGACCGTGACCATTAGGATTG
	P3	ACAATCCTAATGGTCACGGTCA	GCAGACCCTGTGGAATTTGATT
	P4	AGGGTCCTGTAGATCCTCAAAC	TCATCACATAAGGTGCATAGCC
	P5	AAATTCCACAGGGTCTGCTGC	AGGACAACCGTCACGCGATAT
	cDNA	GATTTTAAAATATCAGACTAGAGGCTGA	CAGGATGGCAAGAAACATTTATTC
For qRT-PCR	GTPCH	TGGGGGAGGACCCTGAAAG	CGAAGACGGCGGGAGAATA
	PTPS	CGCAATAGATGTCTGGGACCAAC	GGACGAGAGGAGTCAGGCTTTT
	SPR	GAAATCCGTTATCCTGCTCCTG	TCTGTCTCGGCGATGCCTTACT
	DHFR	GCTTACTTCACGACGATGACAACT	CTAATCTAATCCTGGCACAACAAT
	PAH	CCCTCATACGGTGCCGAACT	CATCAGCAGCGGGAAGACAT
	TH	TTGATGCCCAAACACGC	TCGCAGGGTAAAGCCAGT
	sw22934	TTCGTACTGCTCTTCTCGT	CAAAGTTGATAGCAATTCCCT
